# Object-based representation and analysis of light and electron microscopic volume data using Blender

**DOI:** 10.1186/s12859-015-0652-7

**Published:** 2015-07-25

**Authors:** Albina Asadulina, Markus Conzelmann, Elizabeth A. Williams, Aurora Panzera, Gáspár Jékely

**Affiliations:** Max Planck Institute for Developmental Biology, Spemannstrasse 35, 72076 Tübingen, Germany

**Keywords:** *Platynereis*, Gene expression atlas, Connectome, Surface representation, 3D model, Blender

## Abstract

**Background:**

Rapid improvements in light and electron microscopy imaging techniques and the development of 3D anatomical atlases necessitate new approaches for the visualization and analysis of image data. Pixel-based representations of raw light microscopy data suffer from limitations in the number of channels that can be visualized simultaneously. Complex electron microscopic reconstructions from large tissue volumes are also challenging to visualize and analyze.

**Results:**

Here we exploit the advanced visualization capabilities and flexibility of the open-source platform Blender to visualize and analyze anatomical atlases. We use light-microscopy-based gene expression atlases and electron microscopy connectome volume data from larval stages of the marine annelid *Platynereis dumerilii*. We build object-based larval gene expression atlases in Blender and develop tools for annotation and coexpression analysis. We also represent and analyze connectome data including neuronal reconstructions and underlying synaptic connectivity.

**Conclusions:**

We demonstrate the power and flexibility of Blender for visualizing and exploring complex anatomical atlases. The resources we have developed for *Platynereis* will facilitate data sharing and the standardization of anatomical atlases for this species. The flexibility of Blender, particularly its embedded Python application programming interface, means that our methods can be easily extended to other organisms.

**Electronic supplementary material:**

The online version of this article (doi:10.1186/s12859-015-0652-7) contains supplementary material, which is available to authorized users.

## Background

Recent advances in tissue labeling and light and electron microscopy imaging techniques have greatly improved our ability to acquire volume data from biological specimens. Light microscopic volumes are commonly obtained following specific tissue labeling protocols to highlight transgene expression, gene expression patterns, or immunolabels. The increasing use of anatomical atlases [1–7], where the signal from different individuals is represented in an average anatomical map, poses the challenge of visualizing hundreds of channels simultaneously. Similarly, electron microscopy serial reconstructions can generate large volume data with hundreds of objects that are computationally hard to visualize.

Several general-purpose visualization tools, including Amira [8], Imaris [9], 3D graphics software Maya [10], Blender [11], and 3ds Max [12] are available for image analysis. In addition, many tools are available that were specifically developed for visualizing and analyzing biomedical images, such as VTK [13], ParaView, 3DSlicer [14], InVesalius [15], MIA [16], and Vaa3D [17]. Some software tools, including the Brain Explorer [18], CoCoMac [19], and PointCloudXplore [14], focus on a particular species.

Visualization of connectome data is currently achieved using software tools including 3D View in TrakEM2 [20], 3D Viewport in Knossos [21], Structure Viz in Viking [22] and Rambo3D [23]. Connectome Viewer [24] was designed for neuroimaging data and was applied to Magnetic resonance imaging images. ConnectomeExplorer [25] provides interactive visualization of electron microscopy image stacks together with 3D volumes of the objects reconstructed from that stack.

The number of channels that can be displayed in tools for visualizing light microscopy volume data is often hardware-limited due to the large data sizes inherent to pixel-based representations. For such volumes, segmentation into a binary image, followed by surface representation, is a memory-efficient alternative. Similarly, serial EM reconstructions that outline object borders or neuronal skeletons are binary and can be represented as surfaces or skeletons.

We were searching for a flexible, general-purpose tool that would allow object-based 3D visualization and computational analysis of gene expression patterns for an unlimited number of genes, as well as the visualization and analysis of large connectome datasets. The ideal tool would allow efficient representation of volume data from any source, be open source and supported by a broad community, and would support efficient functional extension.

For these reasons we chose to use the 3D graphics software Blender, a free and open source 3D graphics suite equipped with numerous options for visualization, modeling and animation. Blender is easy to install, memory-efficient, highly flexible and can be extended by its embedded Python application-programming interface (API). Multiple tutorials, extensive documentation and community support forums make Blender easy to adopt in any laboratory. Blender has already been exploited for the visualization and analysis of neuronal reconstructions and functional Magnetic resonance imaging (fMRI) data [26–28].

Here we demonstrate the potential of Blender for visualizing and exploring scientific imaging data using the example of the marine annelid worm *Platynereis dumerilii*, a model organism for evolutionary developmental biology, zooplankton behavior and neuronal connectomics [29–32]. Due to its small and transparent body, the *Platynereis* larva is well-suited for whole-body light microscopic imaging following *in situ* hybridization or other tissue staining protocols. *Platynereis* early larval development is synchronous and stereotypic, following a strict spiral cleavage pattern. This stereotypy enabled the generation of gene expression atlases for several larval stages [33, 34]. The *Platynereis* larva has also been used for serial electron microscopy imaging and the neuronal connectome of its visual circuit has recently been reconstructed [35]. We used Blender to create surface models of gene expression atlases for three *Platynereis* larval stages. Blender enables the simultaneous visualization of gene expression patterns using a surface-based approach, which dramatically increases the number of genes that can be visualized and analyzed simultaneously compared to intensity-based approaches.

We also used Blender to model the neuroanatomy and connectivity of the *Platynereis* visual circuit [35]. We have extended the functionality of Blender to enable the exploration of connectomes. We provide scripts to import TrakEM2 and Catmaid [36] connectome datasets. We have added functionality to query connections and to map parameters of network statistics onto a 3D model. Blender supports standard file formats, enabling transfer of the 3D models to different platforms, including web and Android mobile applications. Due to the flexibility of Blender, our approach can easily be extended to other types of volume data and to other organisms.

## Methods

### Sample preparation and imaging

RNA *in situ* hybridization, immunochemistry, confocal microscopy and image registration methods used for generating the gene expression atlases were performed as previously described [34]. Electron microscopy and neuronal network reconstruction were carried out as previously described [35].

### Modeling of the gene expression atlases

Whole-body confocal scans of *in-situ* hybridization samples of *Platynereis* larvae were registered to a nuclear-stain (4',6-diamidino-2-phenylindole, DAPI) reference, as described previously [34]. For each gene expression pattern, we averaged individual scans of 4-6 larvae. These average gene expression patterns were then filtered (Median 3D, 4 pixel radius) and manually thresholded. The thresholded average gene expression patterns were represented as surfaces using ImageJ 3D Viewer and exported as .obj files. The surfaces were then imported into Blender and broad gene expression domains were smoothed with a Gaussian filter. An average acetylated-tubulin immunostaining signal was also imported into Blender as a surface to provide anatomical landmarks. The 48 h post fertilization (hpf) atlas includes 19 genes (*ChAT*, *dimmed*, *DLamide*, *DOPAbHyd*, *FLamide*, *FMRFamide*, *FVamide*, *L11*, *LYamide*, *MIP*, *Phc*, *RGWamide*, *RYamide*, *Tinman*, *TrpHyd*, *VAChT*, *VGlut*, *WLD*, *YFamide*), the 72 hpf atlas 23 genes (*ChAT*, *dimmed*, *DOPAbHyd*, *FVamide*, *FVRIamide*, *GAD*, *HisDec*, *L11*, *LYamide*, *MIP*, *Phc*, *PitX*, *r-opsin1*, *RGWamide*, *SPY*, *Synapsin*, *Tinman*, *TRPC*, *TrpHyd*, *TyrH*, *VAChT*, *VGlut*, *WLD*) and the 6 dpf atlas 9 genes (*AKH*, *enteropeptidase*, *FMRFamide*, *FVRIamide*, *Legumain*, *MIP*, *RGWamide*, *Subtilisin-1*, *Subtilisin-2*) [37].

### Group-specific queries of the gene expression models

We used the Blender grouping option to assign gene name, functionality and gene types (e.g. neuropeptide precursor, enzymes) to the gene expression domains. Expression domains associated with the same gene, the same gene type or the same functionality were grouped together. The groups can overlap. The names of the gene-type groups start with a hyphen ‘-’, the names of the gene-function groups start with an underscore ‘_’ and gene names can start with any character.

Any Blender object can be extended with a custom property, which can then be linked to the elements in the user interface. The Blender class Group was extended with a custom Boolean property *Visibility*, which enabled showing and hiding groups of objects according to a user query.

### Gene colocalization

Gene colocalization analysis was enabled using a *Boolean* modifier in Blender, which creates a single compound out of two objects using *difference*, *union* or *intersection* operations. For the colocalization analysis we used an *intersection* operation. The expression domains of two user-specified genes are examined for colocalization using the Boolean modifier and the intersection volumes are then displayed. For each gene pair we determine the overlapping regions and define these as new objects. The volumes of these objects are determined using the Blender plugin MeshVolumeTools [38]. The colocalization is determined as a sum of overlapping volumes for each domain of an expression pattern. We defined a coexpression index as the ratio of the volume of the overlapping regions relative to the volume of the gene with the smaller total volume.

### Modeling of neuronal circuitry

Neurons were segmented or traced as skeletons using TrakEM2 or Catmaid. Synapses and cell body positions were also annotated by expert neuroanatomists based on the raw electron microscopy data, using established procedures [20]. The raw traces were exported from TrakEM2 or Catmaid and imported into Blender and parsed. In the Blender model, neuronal cell bodies were approximated with a sphere. The center and radius of the sphere, approximately matching the size and position of the cell nucleus, were defined during tracing in Catmaid. Skeleton nodes were loaded in a graph-like structure (using the Python *dictionary* class). The neurite branches were then reconstructed using a depth-first search graph traversal algorithm [39]. The branches were smoothed using the Non Uniform Rational B-Splines (NURBS) curves function in Blender.

Branches smaller than 50 nodes were trimmed to simplify neuron representations. The synaptic connections were also imported and rendered in Blender as small spheres in their respective anatomical positions. Annotating with synaptic information was enabled using an *ID-property*. ID-property in Blender is attributed to class instances, rather than to the entire class. It is stored in the source file and therefore remains accessible after reopening a project. The imported neurons were extended with *Presynaptic* and *Postsynaptic* ID-properties that contained the respective lists of the pre- and postsynaptic sites for each cell. The *Presynaptic* and *Postsynaptic* ID-properties were also created for each synapse and contained the lists of pre- and postsynaptic sites respectively [37].

### Group specific queries and network queries of the connectome model

The neuronal circuitry model was annotated with anatomical classification, behavior and gene expression information associated with reconstructed networks. The annotation was implemented using the grouping option in Blender. The names of the groups denoting behavior start with an underscore “_”, the names of the groups denoting expressed genes start with a hyphen “-” and the groups denoting anatomical classification start with any other character. Querying across different groups was enabled as described for the gene expression atlas.

We implemented a functionality to query neuronal connectivity information for the neuronal circuitry model using the ID-properties *Postsynaptic* and *Presynaptic*. We enabled queries for the pre- or postsynaptic sites for a cell of interest. Cells are displayed if they are listed in the ID-property of the cell of interest, otherwise they are hidden. To query for up- or downstream circuitry for a cell of interest, we applied the depth-first search graph traversal algorithm [39]. The neuronal network was considered as a directed graph, where neurons are represented by vertices and synapses are represented by directed edges. All upstream (or downstream) cells are highlighted and the remaining cells are hidden. We also implemented queries for synapses of an individual cell, between two cells or between two groups of cells.

### Centrality metric

The model was extended with a function for measuring network centrality and mapping network centrality in the 3D view. In- and out-degree centralities for each element of the neuronal network were calculated as follows:

$$ CD(x)= \deg (x), $$

where deg (*x*) is the number of edges for the node *x*. All edges directed to the node were calculated for in-degree centrality and all edges directed from the node were calculated for out-degree centrality.

In- and out-closeness centralities were calculated for the neurons in the neuronal circuitry as follows:

$$ CC(x)={\displaystyle {\sum}_{y\in G,y\ne x}\left(\frac{1}{d\left(x,y\right)}\right),} $$

where *d* (*x, y*) is a length of the path from the node *x* to the node *y* for out-closeness centrality and a length of the path from *y* to *x* for in-closeness centrality.

In- and out-eigenvector centralities were calculated as follows:

$$ CX(x)=a\kern0.5em \ast \kern0.5em {\displaystyle {\sum}_{y\in G,y\ne x}\left({g}_{x,y}\kern0.5em \ast \kern0.5em CD(y)\right)}, $$

Where *CD* (*y*) is in- or out-degree centrality of the node *y* for in- and out-eigenvector centrality respectively, a is a constant, *g*_*x,y*_ is equal to one if nodes *x* and *y* are connected, otherwise *g*_*x,y*_ is equal to zero.

### Mobile and web application

The 3D models were exported from Blender using standard formats and visualized on different platforms, for example, Android [40] mobile devices. Android applications were developed using an Android Developer Tools Bundle and Eclipse Integrated Development Environment [41]. The Blender models were exported in OBJ format, converted in GD3D format using the FBX converter [42] and then loaded in the mobile application using libGDX [43]. The manipulation of visibility settings was implemented using the material BlendingAttribute. The web applications were developed using the HTML and JavaScript programming languages and the Three.js library [44]. The models were exported from Blender in the .js (javascript) format and were subsequently loaded in the web applications using the Three.js library that enabled 3D visualization. Model manipulation was enabled using the OrbitControls library from Three.js. Material transparency was used to enable the manipulation of the visibility settings of the objects in the applications. Exploring the models using web applications requires WebGL [45] enabled in the web browser [46].

## Results

### Surface models for *Platynereis* larval gene expression atlases

**Fig. 1:**
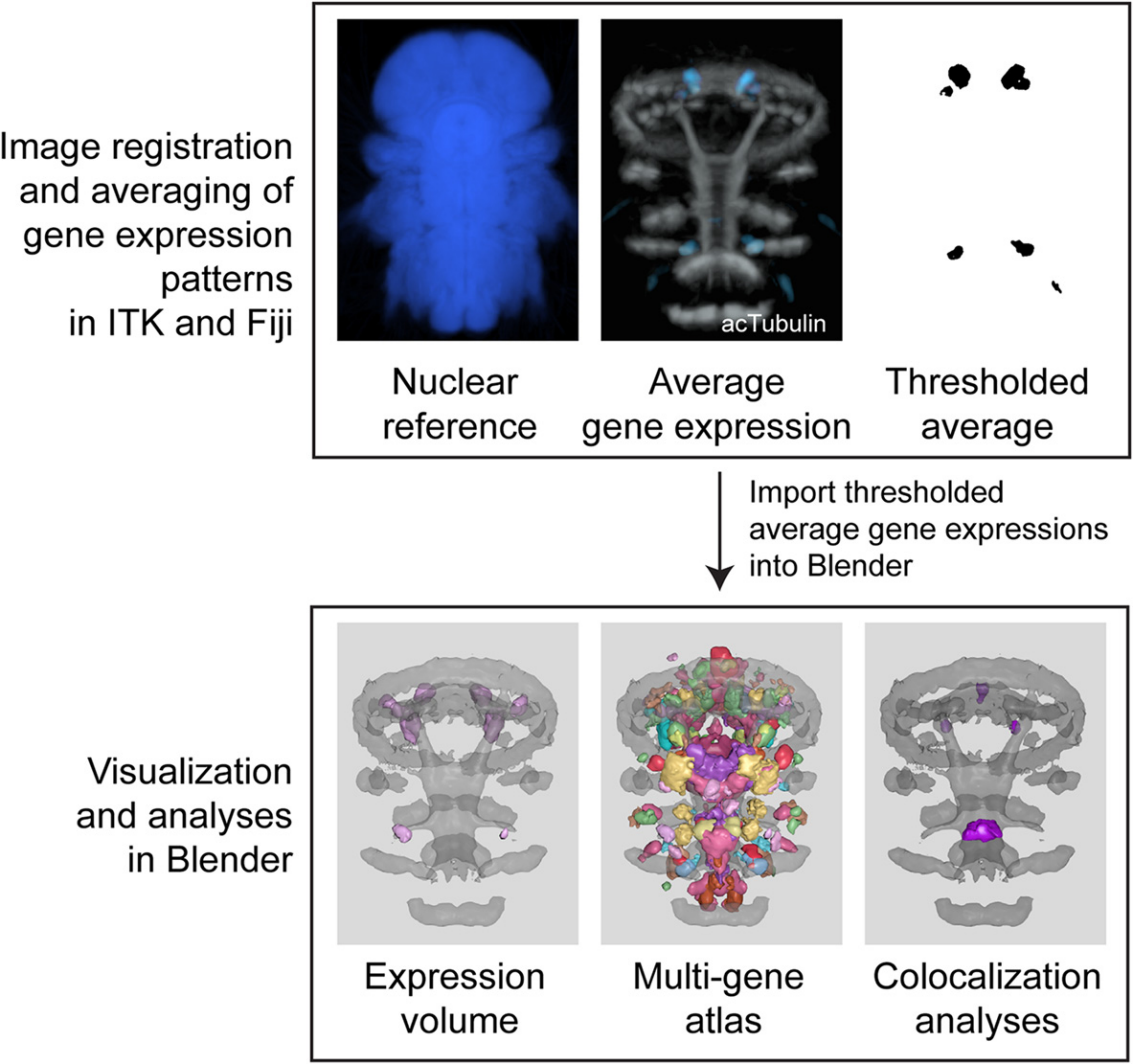
Pipeline for generating a model of a gene expression atlas. An individual gene expression pattern is thresholded and converted into a surface representation using the ImageJ plugin 3D Viewer. The surfaces are then exported from 3D Viewer in OBJ format and imported in Blender. Scale bar 30 μm

**Fig. 2:**
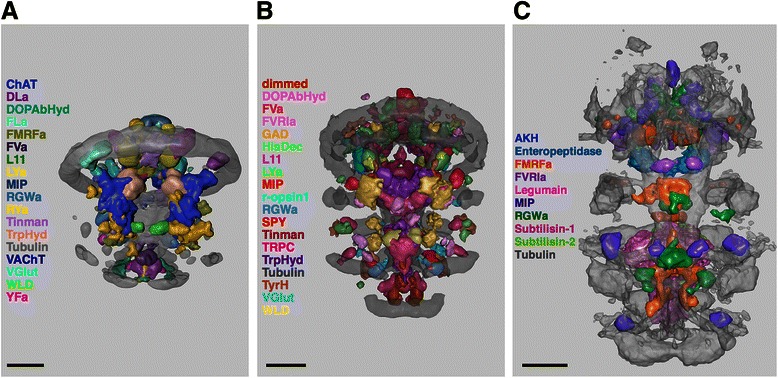
Blender models of gene expression atlases for *Platynereis* larvae. Blender models based on registered and thresholded average gene expression patterns for 48 hpf (**a**), 72 hpf (**b**), 6 dpf (**c**) *Platynereis* larvae. Scale bar 30 μm

*Platynereis* larvae are amenable to near cellular-resolution expression profiling using *in situ* hybridization and gene expression atlases [33, 34]. However, the number of genes that can be simultaneously displayed in an atlas has been limited. We used Blender to efficiently visualize and analyze gene expression atlases for three *Platynereis* larval stages (48 hours post fertilization (hpf), 72 hpf, 6 days post fertilization (dpf)). The atlases were generated by registering gene expression patterns to stage-specific average nuclear-stain (DAPI) templates [34]. For each gene we calculated an average expression pattern, which, following thresholding, was imported into Blender (Fig. 1). To provide an anatomical reference, we also imported an average acetylated tubulin immunostaining signal that labels cilia, axons and dendrites within the larvae. The surface representation used by Blender allows fast 3D visualization for several channels, a feature that is limited in pixel-based visualization methods (Fig. 2).

In the Blender models, the distinct expression domains for each gene are represented as separate objects. This allows the manual curation of the data, for example the annotation of individual cells or expression domains for a gene or the removal of background signal. In *Platynereis* larvae, the spinning glands and parapodial chaetae show a consistent background signal during microscopy. This signal would bias downstream calculations (e.g. analysis of coexpression) and the corresponding domains were therefore manually removed from the gene expression models.

### Annotation of gene expression domains

The surface-based approach, together with the extensive volume analysis tools available in Blender, allowed us to treat gene expression domains as individual objects. These objects can be grouped and annotated. We annotated gene expression domains with gene name, type of gene product (e.g. receptor) and gene function. These annotations allow flexible Python-based queries (attribute queries) and enable the user to hide/show gene expression patterns that share a common annotation (Additional files 1 and 2).

### Exploring gene expression colocalization

**Fig. 3:**
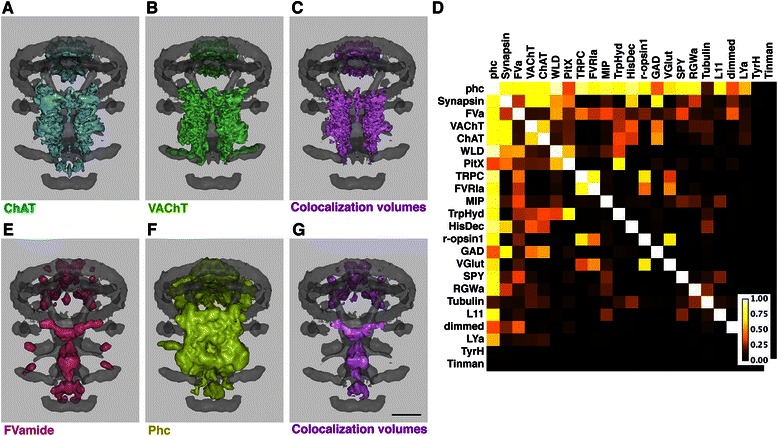
Detection of gene colocalization in the gene expression model. (**a**, **b**, **e**, **f**) modeled gene expression patterns. (**c**, **g**) Colocalization volumes for the selected gene expression models. (**d**) Colocalization matrix for the gene expression models in 72 hpf *Platynereis* larvae. Scale bar 30 μm

Visual inspection for gene expression colocalization for a large number of gene pairs in a gene expression atlas containing dozens or hundreds of genes is a challenging task. To facilitate this task, we provided a function that enables querying colocalization of gene expression patterns in the Blender model (Additional files 1 and 2). For each gene pair in the model we determine the overlapping volumes and define these as a new object (Fig. 3a, b ). Based on the overlap volume and the volume of the two genes we then defined a coexpression index. We also developed a script that determines a coexpression matrix for all gene pairs. We demonstrate this method using the 72 hpf atlas (Fig. 3d). We also measured the performance of the functionalities we implemented in Blender. Importing, colocalization analysis and querying were all completed within the msec-sec range on a standard desktop computer (Table 1).

**Table 1:** Performance of Blender. The performance of the different Blender functionalities was evaluated on an iMac with a 3.06 GHz Intel Core i3 processor and 8 Gb memory

Activity	Time (ms)
*Gene expression atlas model*	
Import gene surface representation	150-600
Find gene colocalization for a pair of genes	200-9000
Query metadata	1-2
*Neuronal network model*	
Import a neuron	150-350
Import muscles (verts:346710 faces:681556)	17600
Import glia (verts:140041 faces:341738)	9200
Metadata query neuronal circuitry	10-30
Find up-/downstream neurons	20-60
Calculate centrality	250-500

### Blender representation of neuronal connectomes

**Fig. 4:**
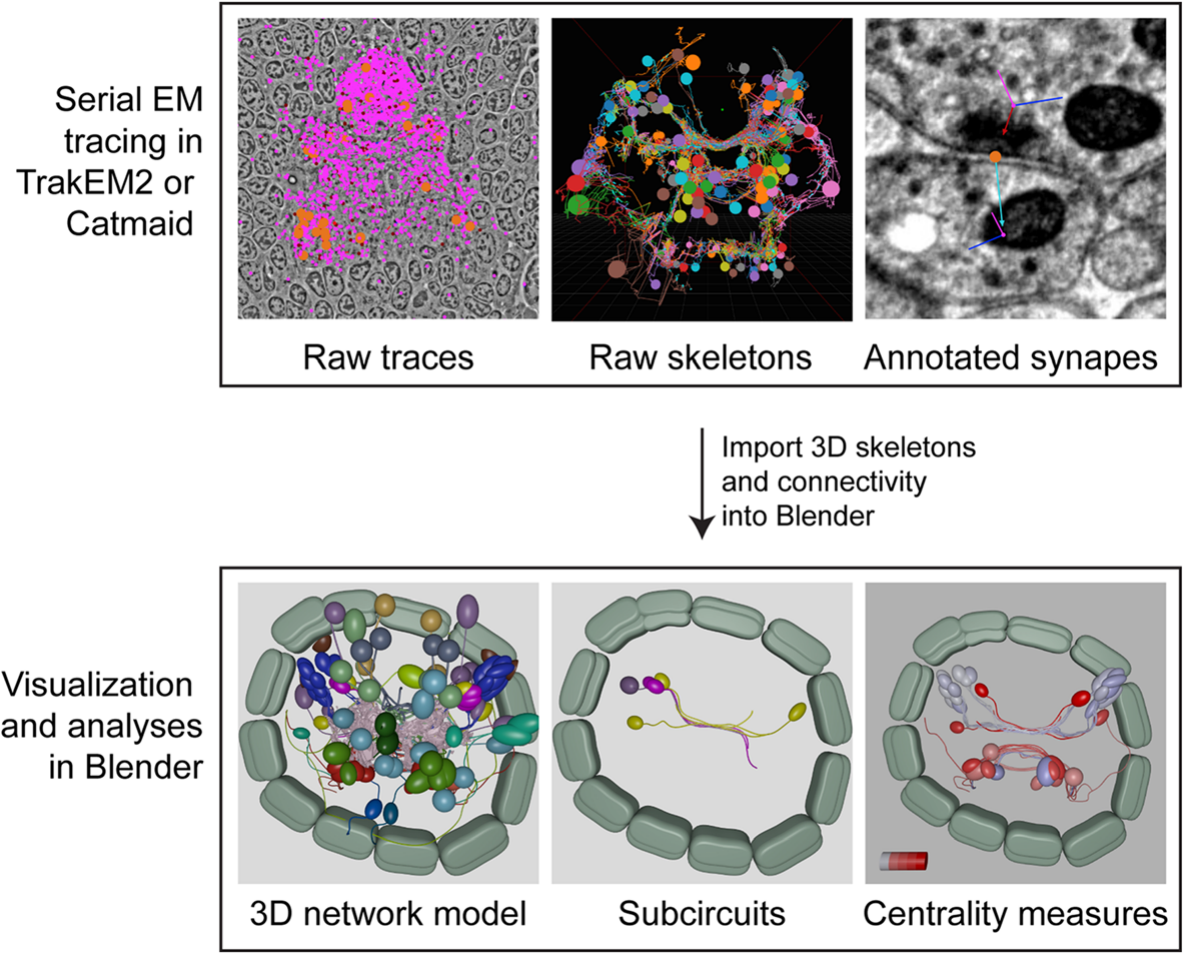
Pipeline for generating a model of a neuronal network. Individual neurons are traced from the original electron microscopy images. The tracings are then exported in OBJ format using 3D Viewer in TrakEM2. The neurons are modeled in Blender using Python scripts. Synaptic connections are subsequently integrated into the model

In addition to developing sophisticated gene expression atlases, the advanced visualization and scripting tools available in Blender allowed us to develop a platform for the visualization and analysis of complex neural connectomes. Neuronal connectome datasets combine complex 3D morphological information with information on underlying neuronal networks. We focused on the visual neuronal network of the *Platynereis* larva, reconstructed from serial-section electron microscopy images of a 72 hpf specimen [35]. We developed this platform further by providing import functionality for TrackEM2 and Catmaid connectome projects and further tools for connectivity analysis (Additional files 1 and 2). In the model, we represented the cell bodies of reconstructed neurons as spheres, and the axons and dendrites as smoothed tubes (Fig. 4). Complex anatomical shapes, including glial cells and muscles, were modeled as surfaces or were manually approximated with built-in geometric shapes (Fig. 4). The simplification of cell morphologies led to a great decrease in file size, facilitating the sharing of the atlases (Table 2). The modeled structures were annotated with anatomical terms (e.g. photoreceptor) and gene expression information (e.g. r-opsin1). These classifications can be further extended by the user.

**Table 2:** Performance of Blender. The performance of the different Blender functionalities on raw and modeled neuronal reconstructions was evaluated on an iMac with a 3.06 GHz Intel Core i3 processor and 8 Gb memory

	Raw reconstructions	Neuron models
File size with 10 neurons	22 Mb	963 Kb; (empty file, 429 Kb)
Rotation of a single cell	0.5 ms	0.2 ms
Hiding a single cell	0.03 ms	0.03 ms

We also imported synaptic connectivity information into the model. Each neuron is automatically annotated with information on its pre- and postsynaptic sites and all synapses are represented as spheres in their corresponding positions in the 3D space. Each synapse is also annotated with information on its pre- and postsynaptic cells.

To explore the neuronal circuitry, we implemented functionalities to query different aspects of the synaptic connections (Additional files 1 and 2). Users can highlight all incoming or outgoing synapses for any neuron and display all synapses between pairs of neurons. In addition, the user can explore signal propagation by highlighting direct pre- and postsynaptic partners or complete down- and upstream circuitry for a neuron of interest.

**Fig. 5:**
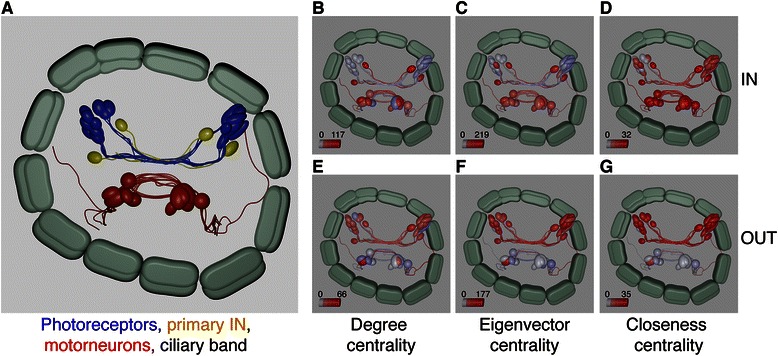
Measuring network centrality in Blender for the *Platynereis* visual neuronal network (**a**). Degree (**b**, **e**), eigenvector (**c**, **f**) and closeness centrality (**d**, **g**) projected onto the 3D model of the neuronal network

Blender also enables the calculation of statistical parameters and their visualization in a 3D model of a neuronal network. We calculated network centrality measures for the *Platynereis* eye connectome including degree, eigenvector and closeness centrality, and mapped these measures onto the 3D neuronal model (Fig. 5, Additional files 1 and 2). These centrality measures reflect various aspects of connectivity of the nodes of a network and can therefore represent information flow or highlight the importance of individual neurons in the network (Fig. 5).

### Interactive Android and web applications for the Blender models

3D models can be exported from Blender in different standard formats (such as OBJ or STL) and subsequently transferred onto different platforms. We exploited this feature to represent our models in a web application using the Three.js library [44]. Model elements (e.g. gene names, anatomical terms, classifications) can also be queried in the application. We also developed interactive Android applications using the libGDX library to visualize Blender models on mobile devices. The models were scaled down to allow their effective visualization. The web and Android applications provided an efficient means of accessing and sharing the models without the need to install Blender.

## Discussion

We exploited the powerful visualization and computational capabilities of the free graphics software Blender for visualizing and analyzing gene expression and connectome volume data. Blender provides functionalities for efficient and high-quality visualization, modeling, rendering and animation of volume data, all of which significantly enhance data representation. The flexible grouping and annotation of objects in Blender enables efficient representation and exploration of the data. For example, the grouping option allows the annotation of models with additional information, the option to extend objects with arbitrary properties allows the user to store synaptic connectivity information in a neuronal circuit. The embedded Python API makes Blender extremely adaptable to specific problems. We used the Python API to develop scripts for importing volume data, querying annotations and connectivity, exploring gene colocalization and calculating network centrality measures.

Since the majority of users have little or no programming skills [47], it is essential that software tools for analyzing biological images have user-friendly interfaces. The installation of Blender is simple, as it is distributed via binaries and can run immediately after downloading. Blender does not require additional libraries or any adjustment of system settings. The models, with their embedded scripts, can be distributed in Blender format and directly opened in Blender. All functionalities required to update the models or create new models are provided in the Blender file.

## Conclusions

Blender is an adaptable and efficient tool for the visualization and analysis of large volume microscopic data. Using volume-imaging data from the annelid *Platynereis*, we demonstrated the flexibility of Blender in performing microscopy data analysis. The presented approach is not restricted to *Platynereis* and can be extended to any other organism.

## Availability of supporting data

The data sets supporting the results of this article are available in the GitHub repository, https://github.com/JekelyLab/BlenderAtlases, https://github.com/albina-a/Web_and_Mobile_applications, and within the article (Additional files 1 and 2).

## Additional files

Additional file 1:
**Instructions for creating and using Blender models.** The file provides instructions for creating and using Blender models of a gene expression atlas or a neuronal network model.
Additional file 2:
**Python scripts for Blender.** The archive provides Python scripts enabling functionality for Blender models.

## Abbreviations

AKH: Adipokinetic hormone
API: Application programming interface
ChAT: Choline acetyltransferase
DAPI: 4',6-diamidino-2-phenylindole
dpf: days post fertilization
DOPAbHyd: Dopamine beta hydroxylase
EM: Electron microscopy
fMRI: Functional Magnetic resonance imaging
GAD: Glutamate decarboxylase
HisDec: Histidine decarboxylase
hpf: Hours post fertilization
HTML: HyperText Markup Language
MIP: Myonihibitory pepetide
Phc: Prohormone convertase
STL: Standard Tessellation Language
TrpHyd: Tryptophan hydroxylase
TyrH: Tyrosine hydroxylase
VGluT: Vesicular glutamate transporter
VTK: Visualization Toolkit.
