# Supplementary material for: Object-based representation and analysis of light and electron microscopic volume data using Blender
Source: BMC Bioinformatics. 2015 Jul 25;16:229. doi: 10.1186/s12859-015-0652-7 (PMC4513682; doi:10.1186/s12859-015-0652-7)
Supplement: Additional file 1: — Instructions for creating and using Blender models. The file provides instructions for creating and using Blender models of a gene expression atlas or a neuronal network model. [file 12859_2015_652_MOESM1_ESM.pdf]

Blender can be downloaded from:
http://www.blender.org/

Gene expression atlas
Explore the gene expression atlases
1. Attribute queries: open and run (ctrl+p) the scripts Panel_LM_genes.py, Panel_LM_behavior.py or Panel_LM_GeneTypes.py in Blender to enable a panel for querying by gene name, gene functionality or gene type respectively. Panels will appear in the 'Tools' menu to set show/hide options for different groups. Press 'apply' to update changes.
2. Coexpression: open and run (ctrl+p) the script Panel_LM_coexpression.py to enable gene coexression queries. Select gene names in the dropboxes and press the 'Show coexpression' button to find coexpression between two genes. To remove the coexpression volumes from the project press 'Remove coexpressions'. To calculate coexpression matrix, start Blender from the terminal and press 'Coexpression matrix' in the 'Gene Coexpression' panel. The matrix will be printed out in the terminal. Copy it into Excel, format and save as a text file. Import as 'Text Image' in ImageJ and adjust the colors.

Create a new model of a gene expression atlas
1. Create a new Blender project
2. Import gene expression patterns in a surface form (we used 3D Viewer in ImageJ/Fiji to generate surfaces). Adjust scale, location and materials. Python API can be used to facilitate adjustments.

Make a project compatible with queries
1. Add gene expression domains to the 'Genes' group
2. Annotate with additional information if available: gene functionality groups start with '_', gene types start with '-' and anatomical groups can start with anything else. This ensures that the groups appear in the corresponding panels.

Access the functionality
1. Import the scripts Panel_LM_genes.py, Panel_LM_behavior.py or Panel_LM_GeneTypes.py into Blender to enable queries by gene name, gene functionality or gene type respectively.
2. Import/open the script Panel_LM_coexpression.py and run it to enable gene coexression queries and calculation of a coexpression matrix.

Neuronal network model

Explore the neuronal network model

1. Attribute queries: open and run the scripts (ctrl+p) Panel_EM_Behavior.py, Panel_EM_Genes.py or Panel_EM_Groups.py in Blender to enable queries for behaviour, expressed genes or anatomical groups respectively. Panels will appear in the 'Tools' menu to set show/hide options for different groups. Press 'apply' button to update the changes.
2. Connectivity queries: open and run (ctrl+p) the script Panel_EM_connectivity.py to enable connectivity queries. Select the cell body of a cell of interest to query for pre- or postsynaptic cells or subnetworks. Select cell bodies of two cells of interest and press 'show connectors' to highlight all synapses from the cell first selected to the cell selected second. Select attribute group names in the dropboxes and press 'show intergroup connections' to show all synapses between two groups.
3. Centrality: open and run (ctrl+p) the script Panel_EM_centrality.py to enable calculation of the centrality metric. The type of centrality can be specified. Afterwards, colors can be reset to the original color-coding.

Create a new neuronal network model

Import neurons

1. Create a new Blender project
2. Open and run the script Panel_EM_import_Catmaid.py or Panel_EM_import_Trakem.py depending on your data source. A panel for import will appear in the 'Tools' menu. Specify an OBJ file name (in the 'neuron file' field) for TrakEM2 import or a *.neuroml file name (in the 'Catmaid file' field) for Catmaid import.
3. After pressing the 'import' button, the neuronal structures will appear in Blender. Edit the structures if necessary and adjust the materials.

Import synapses

Specify a *.neuroml file for Catmaid import or two files for TrakEM2 (connectivity graph and connector coordinates) in the same panel used for the import of neurons. After pressing 'import', synapses will appear in Blender.

Make project compatible with attribute queries

1. Add all neurons into the 'Cells' group (you can use Python scripting here).
2. Add synapses into the 'Connectors' group
3. Add neurons into the 'Neurons' group.
4. Annotate with additional information if available: behavior groups start with '_', gene groups start with '-' and anatomical groups can start with anything else. This ensures that the groups appear in the corresponding panels.

Access the functionality

1. Import the scripts Panel_EM_Behavior.py, Panel_EM_Genes.py or Panel_EM_Groups.py into Blender to enable queries for behaviour, expressed genes or anatomical groups respectively.
2. Import the script Panel_EM_connectivity.py to enable connectivity queries.
3. Import/open the script Panel_EM_centrality.py and run it to enable calculation of the centrality metric.
